# A Systematic Review of Testosterone Therapy in Men With Spinal Cord Injury or Traumatic Brain Injury

**DOI:** 10.7759/cureus.34264

**Published:** 2023-01-27

**Authors:** Ryan J McLoughlin, Zhiye Lu, Amelie C Warneryd, Randel L Swanson

**Affiliations:** 1 Physical Medicine and Rehabilitation, University of Pennsylvania Perelman School of Medicine, Philadelphia, USA; 2 College of Arts and Sciences, University of Pennsylvania, Philadelphia, USA; 3 Center for Neurotrauma, Neurodegeneration and Restoration, Corporal Michael J. Crescenz VA (Veteran Affairs) Medical Center, Philadelphia, USA

**Keywords:** physical medicine and rehabilitation, traumatic brain injury, spinal cord injury, androgen, testosterone

## Abstract

Spinal cord injuries (SCI) and traumatic brain injuries (TBI) increase the risk of testosterone deficiency and result in adverse changes in body composition and poor functional outcomes. The current systematic review aims to provide insights into the use of testosterone therapy for treating men with SCI and TBI. The PubMed and EMBASE databases were systematically reviewed using appropriate terms, and resulting manuscripts were screened using defined Preferred Reporting Items for Systematic Reviews and Meta-Analyses (PRISMA) criteria. The patient population included male patients with SCI or TBI. Further inclusion criteria were: a) human participants 18 years of age or older; b) manuscript published in English; c) study included an intervention with exogenous testosterone; and d) articles published in peer-reviewed journals with full text available. Two reviewers independently extracted data regarding injury type, intervention, and outcomes. Following screening for inclusion/exclusion criteria, a total of 12 primary research studies conducted over the last 30 years were included. Men with SCI were investigated in 11 articles. The combination of testosterone patches and resistance training with functional electrical stimulation (FES) for 16 weeks in men with SCI and an average baseline testosterone level above the cutoff for testosterone deficiency increased muscle mass, strength, bone quality, and basal metabolic rate while testosterone patches without exercise for 16 weeks produced no significant changes in these parameters. Testosterone patches for 12 months in men with SCI and testosterone deficiency also increased lean tissue mass (LTM) and resting energy expenditure (REE). In one study, men with TBI and testosterone deficiency receiving testosterone gel for eight weeks showed a non-statistically significant greater absolute change in functional independence measure (FIM) and grip strength compared to a placebo group. Testosterone therapy with exercise may help improve muscle mass, bone health, strength, energy expenditure, and cardiac health in men with SCI without major side effects. It is difficult to draw conclusions regarding the effects of testosterone therapy in men with TBI based on the limited available evidence. Further investigation is warranted to explore the relationship between testosterone therapy and recovery after SCI and TBI.

## Introduction and background

Spinal cord injuries (SCI) and traumatic brain injuries (TBI) commonly result in significant impairments that limit independent living and increase the risk of chronic medical conditions [1,2]. The rehabilitation of individuals with SCI and TBI requires considerable time and resources, with an estimated individual cost of $300,880 to $634,500 for the first year after SCI and an estimated annual healthcare cost of $40.6 billion in 2016 for nonfatal TBI in the United States [3,4]. Thus, the development of interventions to improve debility and functional outcomes in individuals with SCI and TBI remains of interest.

**Figure 1:**
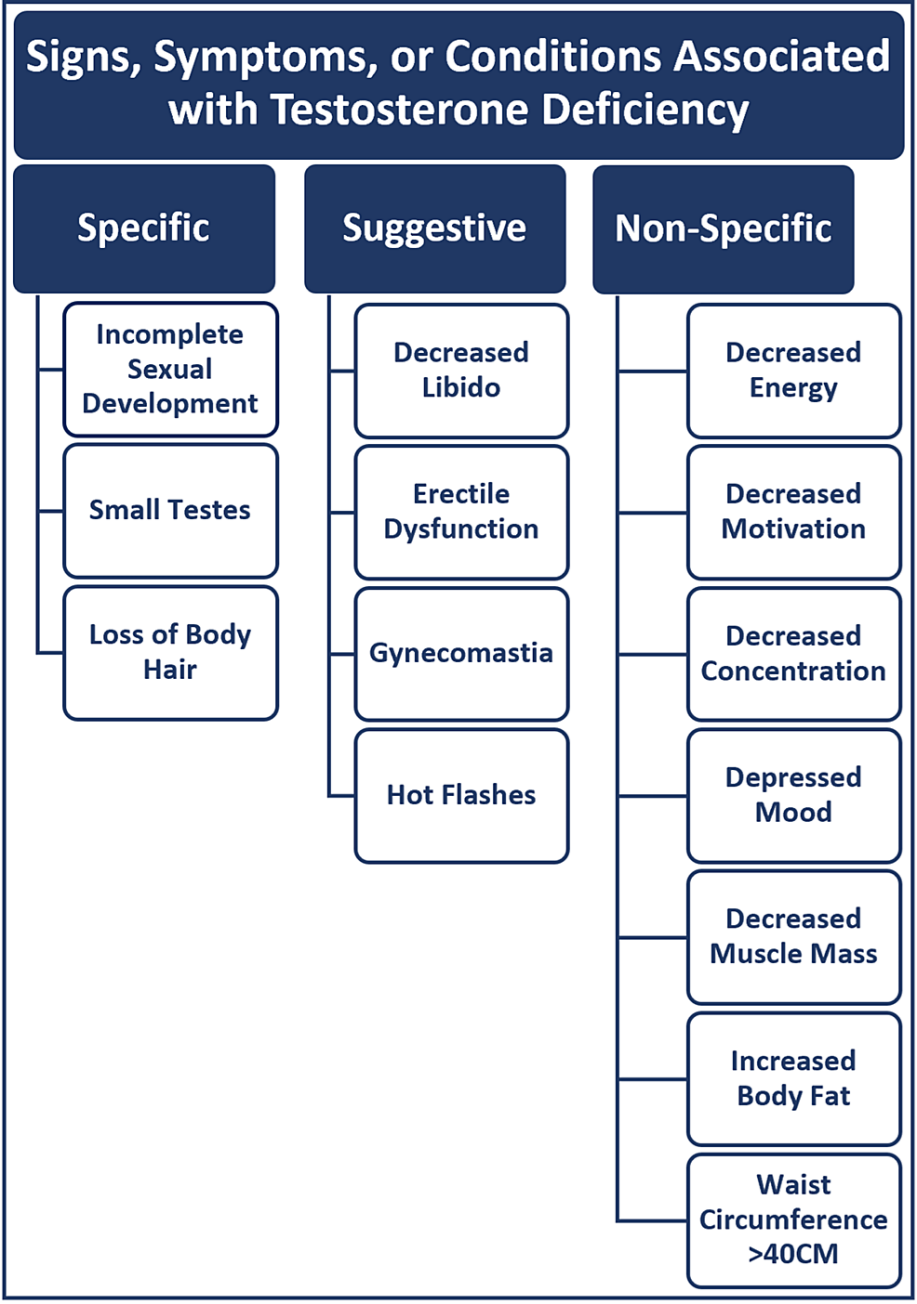
Signs, Symptoms, or Conditions Associated with Testosterone Deficiency

SCI and TBI increase the risk of developing testosterone deficiency [5-9]. Testosterone deficiency, commonly referred to as low testosterone or hypogonadism, is defined as having a morning total testosterone level of less than 300 ng/dl in the setting of signs, symptoms, or conditions associated with testosterone deficiency (Figure 1) [10,11].

A cross-sectional study by Sullivan et al. compared testosterone levels in young men (age < 46 years) with chronic (≥ one year) motor complete (ASIA A or B) SCI without comorbidities to testosterone levels in age-matched men without SCI [5]. Compared to men without SCI, men with SCI had a 3.7-fold higher prevalence of low testosterone with an overall prevalence of 33% based on free testosterone levels. Likewise, other studies have shown an association between SCI and testosterone deficiency [6-8].

Hypopituitarism is a well-documented complication of TBI resulting from both direct mechanical damage and compromised blood flow to the anterior pituitary [12]. Risk factors for the development of hypopituitarism include severe TBI and the presence of imaging findings such as diffuse brain swelling, basal skull fracture, and diffuse axonal injury [13]. While difficult to quantify, the prevalence of hypopituitarism after TBI based on a meta-analysis of 14 studies was reported to be 27.5% [14]. Hypopituitarism is more common in the acute TBI phase and may resolve spontaneously with time. However, testosterone deficiency secondary to hypopituitarism remains common in the chronic TBI phase and guidelines recommend screening for testosterone deficiency one year after TBI exposure [9].

Testosterone deficiency is associated with adverse changes in body composition and worse functional outcomes that may increase the cost of care for those with TBI and SCI [15-17]. Low testosterone in men with TBI admitted for inpatient rehabilitation has been associated with less improvement in functional independence measure (FIM) scores and longer length of stay with an average of an additional 26 days compared to those with normal testosterone levels [16,17]. In men with SCI, Abilmona et al. found that low testosterone was associated with increased fat mass, decreased whole thigh muscle area, and elevated fasting glucose, hemoglobin A1c (HbA1c), and triglycerides [18].

Testosterone therapy is commonly administered through a topical gel, patch, or injectable with a usual dosage range of 50 to 100 mg/day, 2 to 6 mg/day, and 50 to 100 mg/week, respectively [19]. In the general population, testosterone therapy has been shown to improve erectile function, low sex drive, anemia, bone mineral density, lean body mass, and depressive symptoms in men with testosterone deficiency [19]. However, little is known about the benefits of testosterone therapy in patients with SCI or TBI. Therefore, the following systematic review aims to investigate the current evidence of testosterone therapy for treating SCI and TBI with or without testosterone deficiency.

## Review

Methods

Search Strategy

A PubMed and EMBASE search of articles from 1992 to 2022 returned 1075 unique results using the keywords Testosterone or Androgen and Traumatic Brain Injury or Spinal Cord Injury or Rehabilitation Medicine (see Appendix for complete database search criteria).

Eligibility Criteria

Studies were included if they met the following eligibility criteria, established according to the Participant, Interventions, Comparisons, Outcomes, and Study design (PICOS) framework [20].

Participants: Human subjects aged 18 years or older with SCI or TBI

Interventions: Administration of exogenous testosterone

Comparisons: Not applicable

Outcomes: Any objective measure of body composition, physical function, or metabolism

Study design: Studies published between January 1992 and July 2022 and written in the English language were included. Studies must have been published in a peer-reviewed journal with a full text available to review. Review articles, case reports, and perspectives were excluded.

Study Screening

**Figure 2:**
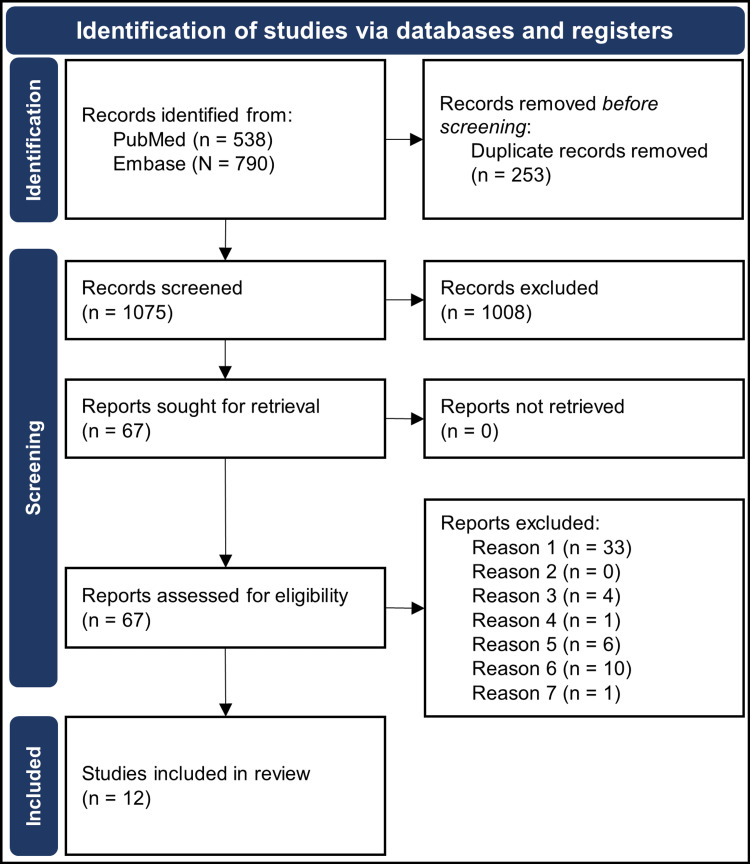
Preferred Reporting Items for Systematic Reviews and Meta-Analyses (PRISMA) Flow Diagram Showing the Selection Process of Reviewed Articles. Defined reasons reports were excluded: Reason one, non-human study; Reason two, participant age < 18; Reason three, intervention without exogenous testosterone; Reason four, participants without spinal cord injury or traumatic brain injury; Reason five, study not published in a peer-reviewed journal with full text available; Reason six, case reports, reviews, or perspectives; Reason seven, non-defined outcome measure(s).

Articles were initially screened based on their titles and abstracts. Two reviewers independently screened the retrieved articles based on the defined PICOS criteria detailed above to create the final list of articles to be included in the review. Screening discrepancies were resolved through a third reviewer. A detailed description of our article selection process is shown in the Preferred Reporting Items for Systematic Reviews and Meta-Analyses (PRISMA) flow diagram below (Figure 2).

Results

Data Abstraction

Two reviewers independently abstracted relevant data from included articles and recorded the data in a predesigned spreadsheet. Demographic information, including author, year of publication, sample size, study design, patient characteristics, and pathology characteristics, was recorded. Data were collected regarding intervention, primary and secondary outcomes, conclusions, and limitations. Details extracted from included studies are presented below, including study characteristics (Table 1), participant characteristics and intervention details (Table 2), study outcomes (Table 3), and study conclusions and major limitations (Table 4).

**Table 1:** Study Characteristics Abbreviations: FES-RT, functional electrical stimulation resistance training; TT, testosterone therapy; SCI, spinal cord injury; LTM, lean tissue mass; REE, resting energy expenditure; HO, heterotopic ossification; CSA, cross-sectional area; TBI, traumatic brain injury; QTaVI, QT apex interval variability.

Study	Study Design	Purpose	Duration	Number of Participants
Holman et al., 2019 [21]	Randomized, open-label clinical trial	To determine whether the combined effects of TT + FES-RT maximize the beneficial effects on muscle quality after SCI.	16 weeks	22
Gorgey et al., 2020 [22]	Secondary analysis of a randomized, open-label clinical trial	To determine the effects of knee extension training with TT + FES-RT on untrained trunk, glutei, & lower leg muscle hypertrophy after motor complete SCI compared to TT alone.	16 weeks	22
Gorgey et al., 2020 [23]	Randomized, open-label clinical trial	To determine the effect of TT or TT + FES-RT on protein expression of key metabolic & hypertrophy regulators, muscle fibers CSA, & markers of mitochondrial health after SCI.	16 weeks	22
Bauman et al., 2011 [24]	Open-label clinical trial	To determine whether 12 months of TT increases LTM & REE in hypogonadal males with SCI.	12 months	22
Bauman et al., 2015 [25]	Open-label clinical trial	To determine whether favorable changes to LTM, REE, & T that occurred after 12 months of TT were retained 6 months after the discontinuation of TT.	18 months	24
Gorgey et al., 2021 [26]	Randomized, open-label clinical trial	To determine the effects of 16 weeks of dose de-escalation with TT + FES-RT on parameters of body composition, cardiometabolic profiles, & neuromuscular parameters compared to no treatment (no TT or FES-RT) in men with chronic complete SCI.	16 weeks	13
Clark et al., 2008 [27]	Retrospective cohort study	To determine whether TT improves motor function in men with SCI compared to an untreated comparison group.	Variable (median of 44.5 days in the control group & 30 days in the intervention group	530
Ripley et al., 2020 [28]	Randomized, double-blind, placebo-controlled pilot trial	To determine the feasibility, safety, & efficacy of TT in hypogonadal men following TBI in acute rehabilitation.	8 weeks	35
Holman et al., 2021 [29]	Randomized, open-label clinical trial	To determine the effects of TT + FES-RT on bony & non-contractile soft tissue.	16 weeks	20
Moore et al., 2016 [30]	Case series	To determine the effects of TT or TT + FES-RT on body composition & HO size after SCI.	16 weeks	2
Gorgey et al., 2019 [31]	Randomized, open-label clinical trial	To determine the effects of low-dose TT + FES-RT on body composition & metabolic variables after SCI.	16 weeks	22
La Fountaine et al., 2013 [32]	Open-label clinical trial	To determine the effect of TT on QTaVI in hypogonadal men with SCI.	12 months	24

**Table 2:** Participant Characteristics and Intervention Details Abbreviations: ASIA, American Spinal Injury Association; FES, functional electrical stimulation; FES-RT, functional electrical stimulation resistance training; IM, intramuscular; lbs., pounds; T, testosterone; TT, testosterone therapy; SCI, spinal cord injury; HO, heterotopic ossification; TBI, traumatic brain injury.

Study	Participant Characteristics (age & mean baseline testosterone levels)	Pathology Characteristics (Type & Chronicity)	Intervention	Control
Holman et al., 2019 [21]	18-50 years; 414 ng/dl	SCI ≥ 1 year; ASIA A or B	T patch 2-6 mg/day based on baseline T plus twice-weekly knee extensions with FES for four sets of 10 reps	T patch alone
Gorgey et al., 2020 [22]	18-50 years; 414 ng/dl	SCI ≥ 1 year; ASIA A or B	T patch 2-6 mg/day based on baseline T plus twice-weekly knee extensions with FES for four sets of 10 reps	T patch alone
Gorgey et al., 2020 [23]	18-50 years; 414 ng/dl	SCI ≥ 1 year; ASIA A or B	T patch 2-6 mg/day based on baseline T plus twice-weekly knee extensions with FES for four sets of 10 reps	T patch alone
Bauman et al., 2011 [24]	18-65 years; Control: 516 ng/dl; Treatment: 257 ng/dl	SCI ≥ 1 year; Non-ambulatory; ASIA A, B, or C	T patch 5 mg/day; If serum T < normal physiologic range after two months then the dose increased to 10 mg/day	Eugonadal group
Bauman et al., 2015 [25]	18-65 years; Treatment: 283 ng/dl; Control: 462 ng/dl	SCI ≥ 1 year; Non-ambulatory; ASIA A, B, or C	After 12 months of T patch 5 or 10 mg/day, TT was discontinued & subjects were followed for an additional six months for re-evaluation	Eugonadal group without treatment
Gorgey et al., 2021 [26]	18-50 years; TT + FES-RT: 171 ng/dl; no-TT: 203 ng/dl	SCI ≥ 1 year; ASIA A or B	T patch 2 mg/day after 16 weeks of T patch 2-6 mg/day plus weekly knee extensions with FES starting with max ankle weight then decreasing 2 lbs. weekly until ankle weight maintained at 2 lbs	No intervention after 16 weeks of the T patch
Clark et al., 2008 [27]	20-56 years; 136 ng/dl	SCI < 1 year; Hypogonadal; Admitted to inpatient rehab	Testosterone cypionate 200 mg IM monthly	National SCI database with age-matched men admitted to rehab over the same time period
Ripley et al., 2020 [28]	18-65 years; 267 ng/dl	Moderate to severe TBI < six months admitted to inpatient rehab	T gel 1% 50 mg/day with dose adjustment after monitoring	Hypogonadal group with placebo gel & eugonadal group without treatment
Holman et al., 2021 [29]	18-50 years; TT: 418 ng/dl; TT + FES-RT: 422 ng/dl	SCI ≥ 1 year; ASIA A or B	T patch 2-6 mg/day based on baseline T plus twice-weekly knee extensions with FES for four sets of 10 reps	T patch alone
Moore et al., 2016 [30]	Ages 31 & 49 years; Not reported	SCI ≥ 1 year; ASIA A or B; History of HO	T patch 2-6 mg/day based on baseline T plus twice-weekly knee extensions with FES for four sets of 10 reps	T patch alone
Gorgey et al., 2019 [31]	18-50 years; 414 ng/dl	SCI ≥ 1 year; ASIA A or B	T patch 2-6 mg/day based on baseline T plus twice-weekly knee extensions with FES for four sets of 10 reps	T patch alone
La Fountaine et al., 2013 [32]	18-65 years; Hypogonadal: 239 ng/dl; Eugonadal: 530 ng/dl	SCI ≥ 1 year; Non-ambulatory; ASIA A, B, or C	T patch 5 mg/day; If serum T < normal physiologic range after 2 months then dose increased to 10 mg/day	Eugonadal group; No intervention

**Table 3:** Study Outcomes Abbreviations: ASIA, American Spinal Injury Association; FES-RT, functional electrical stimulation resistance training; T, testosterone; TT, testosterone therapy; SCI, spinal cord injury; LTM, lean tissue mass; REE, resting energy expenditure; HO, heterotopic ossification; CSA, cross-sectional area; FIM, functional independence measure; PSA, prostate-specific antigen; GLUT4, glucose transporter type 4; Akt, protein kinase B; PGC-1α, peroxisome proliferator-activated receptor gamma coactivator 1-alpha; FAK, focal adhesion kinase; lbs., pounds; TBI, traumatic brain injury; QTaVI, QT apex interval variability.

Study	Primary Outcome	Secondary Outcomes
Holman et al., 2019 [21]	TT + FES-RT improved knee isometric torque by 48.4%, knee extensor CSA by 30.8%, rise time by 17.7% with no significant changes seen in the TT alone group.	TT + FES-RT did not change the half-time to relaxation & increased calcium reuptake by 7%.
Gorgey et al., 2020 [22]	TT + FES-RT for 16 weeks significantly increased the total gluteus maximus & medius muscle CSA area by 14% & 10%, respectively, compared to TT alone.	No significant change in the CSA of trunk & lower-leg muscles between groups.
Gorgey et al., 2020 [23]	TT + FES-RT increased the expression of GLUT4, total Akt, phosphorylated Akt, & mitochondrial activity of succinate dehydrogenase & citrate synthase compared to TT alone. TT + FES-RT increased muscle CSA by 27.5% & TT decreased muscle CSA by 9%.	There was a 27% non-significant increase in serum T in the TT + FES-RT group. TT + FES-RT & TT alone increased PGC-1α & FAK.
Bauman et al., 2011 [24]	TT for 12 months significantly increased total body, trunk, arm, & leg LTM by 7-10% as well as REE by 9%.	No significant change in weight, fat tissue mass, or oxygen consumption.
Bauman et al., 2015 [25]	The significant increase in total body LTM & REE at the end of 12 months of TT in the hypogonadal group was retained after the 6 months of TT discontinuation despite a decrease in serum T levels back to baseline levels.	No significant change in fat tissue mass in both groups. Significantly increased HDL-C levels were maintained in the hypogonadal group. The hepatic panel, hemoglobin, hematocrit, & PSA values were within normal range & did not change significantly in either group over the course of the study.
Gorgey et al., 2021 [26]	Low dose TT + weekly FES-RT for 16 weeks maintained muscle mass & basal metabolic rate compared to the no TT group. Both groups increased visceral adipose tissue without changes in cardiovascular, metabolism, or inflammatory biomarkers.	Low dose TT + weekly FES-RT maintained knee peak isometric & isokinetic torques. The decrease & cessation of TT increased the endogenous T levels.
Clark et al., 2008 [27]	ASIA discharge motor scores for ASIA C & D patients were significantly greater in the TT group compared to the control.	No significant difference in discharge FIM score for men with incomplete or complete SCI. No significant difference in ASIA discharge motor score in complete SCI.
Ripley et al., 2020 [28]	No significant difference between groups in the rate of improvement on the FIM. TT group improved its FIM score by 30 while the placebo group & eugonadal group improved FIM by 19.5 & 17.5, respectively. TT group improved grip strength by 19.5 lbs. while the placebo & eugonadal group improved grip strength by 14.8 lbs. & 5.5 lbs., respectively.	TT did not result in worsening agitation. The percentage of time with agitation or aggression was highest in the placebo group with agitation inversely correlated with T levels. No difference in adverse events between groups.
Holman et al., 2021 [29]	TT + FES-RT for 16 weeks slightly decreased yellow marrow, slightly increased red marrow, & improved trabecular measures at the knee joint. TT alone increased yellow marrow.	TT + FES-RT displayed greater increases in intermuscular fascia length than the TT alone group.
Moore et al., 2016 [30]	TT + FES-RT for 16 weeks increased whole thigh skeletal muscle CSA by 10% & knee extensor CSA by 17% without stimulating the growth of pre-existing HO.	TT alone increased whole thigh skeletal muscle CSA by 13% & knee extensor CSA by 7% without stimulating the growth of pre-existing HO.
Gorgey et al., 2019 [31]	TT + FES-RT for 16 weeks significantly increased total body LTM, whole muscle, & whole muscle knee extensor CSA with no changes in the TT alone group.	Glucose effectiveness improved by 28.5-31.5% in both groups. BMR increased 14-17% in the TT + FES-RT group.
La Fountaine et al., 2013 [32]	QTaVI is significantly elevated in the hypogonadal group at baseline compared to the eugonadal group. TT for 12 months improved QTaVI in hypogonadal men with chronic SCI.	No significant group differences in most of the resting ECG data at baseline or at 12 months. No group difference or intervention effects on lipids.

**Table 4:** Study Conclusions and Major Limitations Abbreviations: FES-RT, functional electrical stimulation resistance training; T, testosterone; TT, testosterone therapy; SCI, spinal cord injury; LTM, lean tissue mass; BMR, basal metabolic rate; REE, resting energy expenditure; HO, heterotopic ossification; GLUT4, glucose transporter type 4; Akt, protein kinase B; CSA, cross-sectional area; FIM, functional independence measure; TBI, traumatic brain injury; QTaVI, QT apex interval variability.

Study	Conclusion	Major Limitations
Holman et al., 2019 [21]	TT + FES-RT improved muscle size & contractile mechanics in men with SCI compared to TT alone.	Does not include a group of FES-RT alone for additional comparisons. Mean baseline T levels above the cutoff for T deficiency.
Gorgey et al., 2020 [22]	TT + FES-RT increases muscle hypertrophy in untrained muscles. TT may need to be combined with exercise to induce muscle hypertrophy after SCI.	Does not include a group of FES-RT alone for additional comparisons. Mean baseline T levels above the cutoff for testosterone deficiency.
Gorgey et al., 2020 [23]	Compared to TT alone, TT + FES-RT in men with chronic SCI increased the enzyme Akt that likely contributes to muscle hypertrophy & increased the enzyme GLUT4 that may improve insulin sensitivity. Low-dose TT may not have a meaningful impact on SCI patients with normal baseline T levels.	Did not significantly increase T levels in TT + FES-RT group & did not measure post-intervention T levels in the TT alone group. Does not include FES-RT alone group. Mean baseline T levels above the cutoff for T deficiency.
Bauman et al., 2011 [24]	TT improves LTM & energy expenditure in hypogonadal men with chronic SCI without adversely affecting prostate health or metabolic parameters.	Functional improvement not assessed.
Bauman et al., 2015 [25]	Discontinuation of TT in hypogonadal men with chronic SCI resulted in the return of serum T to baseline with retention of LTM & REE improvement.	Limited sample size & inherent diversity of the study sample limited ability to detect a reduction in fat mass. Functional improvement not assessed.
Gorgey et al., 2021 [26]	Low-dose TT + weekly FES-RT prevents deconditioning in men with SCI.	Did not account for dietary habits. Knee peak isometric & isokinetic torques were not measured in the no TT group.
Clark et al., 2008 [27]	TT may improve motor function & strength in men with residual motor function after incomplete SCI but not in men with complete SCI.	Notable baseline difference regarding racial composition & length of stay. Not randomized. The study assumed that a proportion of the comparison group had low T & did not receive TT.
Ripley et al., 2020 [28]	TT is safe & well tolerated in patients with severe TBI with fewer reported adverse events than in the hypogonadal placebo group. Although there were no significant differences in the rate of recovery, the TT group showed the greatest absolute FIM & grip strength improvement.	A small sample size for analysis design. Difficulty with subject enrollment, leading to unequal sample baseline. The severity of TBI did not allow cognitive function assessment.
Holman et al., 2021 [29]	TT + FES-RT likely benefits bony & non-contractile soft tissue health below the level of injury in men with SCI. An increase in intermuscular fascia length may represent an expansion of connective tissue to accommodate the increase in muscle size.	Short duration & frequency of exercise training. Did not evaluate trabecular bone in TT alone group. Mean baseline testosterone levels above the cutoff for testosterone deficiency.
Moore et al., 2016 [30]	TT + FES-RT & TT alone increased muscle size without stimulating the growth of pre-existing HO in 2 men with SCI.	Case series limited to two participants is difficult to generalize. The study duration might be too short to allow changes in bone structure.
Gorgey et al., 2019 [31]	TT + FES-RT increases BMR & muscle size in men with SCI compared to TT alone.	Does not include a group of FES-RT alone for additional comparisons. Mean baseline T levels above the cutoff for T deficiency.
La Fountaine et al., 2013 [32]	TT for 12 months in hypogonadal men with chronic SCI improves QTaVI, suggesting a reduction in risk for arrhythmia.	The absence of a matched able-bodied control group limits generalization beyond men with SCI.

Study Characteristics

A total of 12 articles were found to meet our PICOS criteria and were a part of this review, including 10 clinical trials, one case series, and one retrospective cohort study. Patients with SCI were studied in 11 articles while patients with TBI were studied in one article. Chronic SCI, defined as an injury older than one year, was explored in 10 out of the 11 articles. The duration of clinical trials ranged from eight weeks to 18 months and the number of participants ranged from 13 to 35. Participant age ranged from 18 to 65 years. Testosterone patches were investigated in 10 articles while one article studied testosterone gel and one article looked at testosterone injections.

Skeletal Muscle

The combination of a testosterone patch 2-6 mg/day and twice weekly knee extensions with functional electrical stimulation (FES) performed four times for 10 repetitions for 16 weeks in men with chronic SCI significantly increased knee extensor muscle cross-sectional area by 30.8% [21] and the untrained gluteus maximus and medius muscle cross-sectional area by 14% and 10%, respectively [22]. This was associated with an increase in total and phosphorylated protein kinase B (Akt), which likely contributes to muscle hypertrophy [23]. Conversely, no significant changes were seen in the testosterone patch without exercise group.

Similarly, 12 months of a testosterone patch of 5-10 mg/day in men with chronic SCI and testosterone deficiency with the maintenance of their usual dietary and physical activity significantly increased total body, trunk, arm, and leg lean tissue mass by 7-10% [24]. Interestingly, these changes were retained six months after the discontinuation of testosterone therapy despite a decrease in serum testosterone levels back to baseline levels [25].

Strength

The combination of a testosterone patch 2-6 mg/day and twice weekly knee extensions with FES performed four times for 10 repetitions for 16 weeks in men with chronic spinal cord injuries also significantly increased knee isometric torque by 48.4% with no significant changes seen in the testosterone therapy without exercise group [21]. These strength changes were maintained in a group receiving a low-dose testosterone patch of 2 mg/day along with once-weekly knee extensions with FES for an additional 16 weeks [26].

In a retrospective cohort study [27], Clark et al. found that monthly testosterone cypionate 200 mg injections in men with American Spinal Injury Association (ASIA) C and D SCI admitted to inpatient rehabilitation had significantly greater ASIA discharge motor scores compared to a national SCI database that included men in the same age range admitted to inpatient rehabilitation over the same time period. There was no significant difference in ASIA discharge motor scores in patients with complete (ASIA A) SCI [27].

Testosterone gel 50 mg/day for eight weeks in men with testosterone deficiency and moderate-to-severe TBI less than six months post-TBI exposure improved grip strength by 19.5 pounds while placebo gel in men with TBI and testosterone deficiency only increased grip strength by 5.5 pounds. In comparison, the eugonadal group increased grip strength by 14.8 pounds [28].

Functional Assessment

Two studies evaluated changes in the functional independence measure (FIM) score with testosterone therapy [27,28]. Ripley et al. found that testosterone gel 50 mg/day for eight weeks in men with testosterone deficiency and moderate-to-severe TBI did not significantly change the rate of FIM score improvement [28]. However, the testosterone-deficient group receiving testosterone therapy improved the FIM score by 30 while the testosterone-deficient group receiving placebo gel and the eugonadal group improved the FIM score by 19.5 and 17.5, respectively. Additionally, testosterone therapy did not result in worsening agitation, and the percentage of time with agitation or aggression was highest in the placebo group, with agitation inversely correlated with testosterone levels. A retrospective analysis of SCI patients admitted to inpatient rehabilitation revealed no significant difference in discharge FIM score between men with SCI receiving testosterone therapy and the comparison group [27].

Bone

Testosterone patches with twice weekly knee extensions with FES for 16 weeks in men with chronic SCI increased proximal tibial plate width, trabecular thickness, and network area while decreasing trabecular spacing and spacing variability with medium effects and decreased yellow bone marrow with a small effect [29]. A case series of two men with chronic SCI and a history of heterotopic ossification (HO) receiving testosterone patches for 16 weeks showed an increase in muscle size without stimulating the growth of pre-existing HO [30].

Metabolism

Three articles studied changes in energy expenditure [24,25,31]. Bauman et al. showed that 12 months of a testosterone patch of 5-10 mg/day in men with chronic SCI and testosterone deficiency with the maintenance of their usual dietary and physical activity significantly increased resting energy expenditure by 9% with no change in the eugonadal group [24]. These changes were maintained after six months of testosterone therapy discontinuation despite a decrease in serum testosterone levels back to baseline levels [25]. Similarly, the combination of the testosterone patch and FES exercise increased basal metabolic rate by 14-17% with no changes in the testosterone patch without exercise group [31]. Additionally, compared to testosterone patches without exercise, testosterone patches with FES exercise increased the expression of glucose transporter type four (GLUT4), which may improve insulin sensitivity [23].

Cardiovascular System

Fountaine et al. showed that men with SCI and testosterone deficiency have significantly elevated QT apex interval variability (QTaVI) compared to eugonadal men with SCI, suggesting an increased risk for arrhythmia with testosterone deficiency [32]. A testosterone patch of 5-10 mg/day in the testosterone deficiency group for 12 months significantly improved the QTaVI in the men, suggesting a potential mechanism for decreasing the risk of cardiac arrhythmias [32].

Discussion

Skeletal Muscle, Strength, and Bone

Testosterone therapy in combination with an exercise program appears to increase muscle size and strength in men with both complete and incomplete SCI [21,22,24,26,27]. Interestingly, testosterone patches with FES exercise increased both trained and untrained muscle size in men with motor complete (ASIA A or B) SCI [21,22,31]. Testosterone patches without FES exercise in men with ASIA A or B SCI did not change muscle size or strength [21]. However, it is worth noting that the mean baseline testosterone levels did not meet the criteria for testosterone deficiency. Additionally, the testosterone patches did not significantly increase testosterone levels, suggesting that endogenous testosterone levels dropped in response to the testosterone treatment [23]. It is also difficult to say if FES exercise without testosterone therapy would produce similar changes in muscle and strength. While Bauman et al. did not include an exercise routine in their protocol, seven out of the 11 participants in the treatment group engaged in weekly exercise sessions, which likely contributed to the increased lean tissue mass [24].

The lack of statistically significant improvement in grip strength seen with testosterone gel in men with testosterone deficiency and moderate-to-severe TBI admitted to inpatient rehabilitation was limited by the low sample size with unequal baseline grip strength between the treatment and placebo groups [28]. Additionally, the study was conducted over eight weeks, which may not have allowed enough time to produce significant differences between the treatment and placebo groups.

In terms of the evaluation of bone health with testosterone therapy in men with SCI, the researchers did not account for baseline vitamin D and calcium levels. Additionally, trabecular bone was not assessed in the testosterone patches without exercise group due to budgetary constraints but showed an increase in yellow bone marrow [29].

Functional Assessment

Testosterone therapy in the acute inpatient rehabilitation setting may or may not improve FIM scores in patients with SCI and TBI. While Clark et al. found no significant improvement in discharge FIM scores in men with SCI receiving testosterone therapy, the retrospective analysis was limited by notable differences regarding length of stay and assumed that a proportion of the comparison group had low testosterone and did not receive testosterone therapy [27]. Furthermore, Ripley et al. may have found a significant improvement in FIM score in men with TBI receiving testosterone therapy with a larger sample size and longer duration [28].

Metabolism and Cardiovascular Health

Testosterone deficiency is a risk factor for cardiovascular and metabolic disease [19]. Men with testosterone deficiency have an increased risk of myocardial infarction, stroke, hypertension, dyslipidemia, diabetes, and obesity [19]. It remains unclear whether testosterone therapy decreases the risk of developing cardiovascular disease or diabetes. However, 12 months of testosterone therapy in men with testosterone deficiency both increased resting energy expenditure and improved the QTaVI, suggesting that testosterone therapy may decrease the risk for cardiac arrhythmias and metabolic disease [24,32], though further long-term studies are needed to evaluate this relationship.

Side Effects and Contraindications

Testosterone therapy was generally well-tolerated without major adverse events. Specifically, Bauman et al. saw no abnormal prostate findings on the digital rectal exam and no significant changes in the prostate-specific antigen (PSA), liver function tests, mood, and lipid profile after 12 months of testosterone therapy, which was the longest duration of testosterone therapy studied [24]. However, the average age of the treatment group was 43 years with a standard deviation of six years, which limits generalizations that can be made to older men taking testosterone therapy. They also excluded participants with a history of prostate cancer or current elevated PSA greater than or equal to 4 ng/ml, elevated hematocrit greater or equal to 55%, abnormal liver function tests, abnormal digital rectal examination, heart and/or vascular disease, acute or chronic illness of any etiology, cancer, significant psychological and/or sleep disorders. Worth mentioning, the Endocrine Society guidelines do not recommend testosterone therapy in men with prostate cancer, breast cancer, PSA greater than 4 ng/ml or greater than 3 ng/ml in individuals at high risk for prostate cancer, hematocrit greater than 54%, uncontrolled congestive heart failure, history of myocardial infarction or stroke within the last six months, severe lower urinary tract symptoms, uncontrolled obstructive sleep apnea, or desire for fertility in the near term [33].

Future Studies

An ongoing randomized, placebo-controlled trial is investigating the combination of testosterone injection and FES exercise versus FES exercise and placebo injection in men and women with SCI [34]. The study should help determine if testosterone therapy provides additional benefits to an exercise program for those with SCI.

Limitations

The major limitations of each individual study included in this review are detailed in Table 4. Collectively, the study duration and sample size were limited in most studies. The duration of nine studies did not exceed 16 weeks in duration, and the number of participants in all of the clinical trials was limited to 35 or fewer participants. This may not allow enough time to develop appreciable changes in body composition. Only one study included a placebo group for comparison [28]. The two studies looking at functional outcome measures did not exceed eight weeks [27,28].

## Conclusions

SCI and TBI increase the risk for a testosterone deficiency and result in adverse changes in body composition and poor functional outcomes. Testosterone therapy with exercise may help improve muscle mass, bone health, strength, energy expenditure, and cardiac health in men with SCI without major adverse effects. It is difficult to draw conclusions regarding the effects of testosterone therapy in men with TBI based on the limited available evidence. However, findings from a small clinical trial suggest that testosterone therapy with rehabilitation may improve strength and FIM scores in men with TBI and testosterone deficiency compared to rehabilitation alone. Further investigation is warranted to explore the relationship between testosterone therapy and recovery after SCI and TBI.

## Figures and Tables

**Figure 3:**
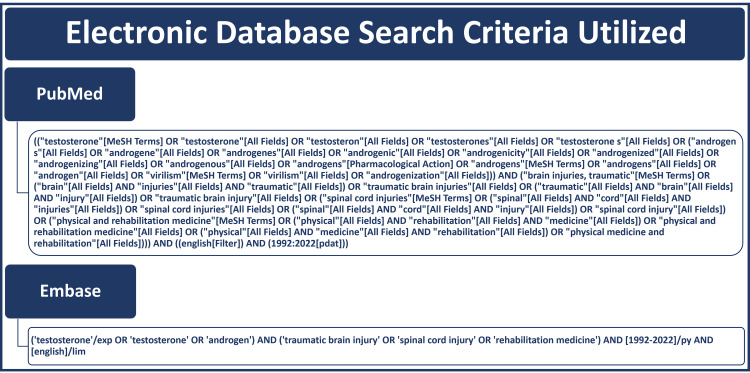
Electronic Search Criteria Abbreviations: MeSH, medical subject headings

## Appendices

Supplemental material

Complete database search terms for both PubMed and Embase are displayed below (Figure 3).
